# Participation of the caudal cerebellar lobule IX to the dorsal attentional network

**DOI:** 10.1186/s40673-018-0088-8

**Published:** 2018-06-15

**Authors:** Ramanoel Stephen, York Elizabeth, Habas Christophe

**Affiliations:** 1 Institut de la Vision (CHNO des 15-20), CNRS, INSERM, Université Pierre et Marie Curie, 28, rue de Charenton, 75012 Paris, France; 2 Service de NeuroImagerie, CHNO des 15-20, Paris, France

**Keywords:** Cerebellum, Dendate nucleus, Tonsilla, Dorsal attentional network, Ocular saccade, Resting-state, fMRI

## Abstract

**Background:**

We seeked for specific cerebellar contribution within the dorsal attentional network (DAN), using Independent Component Analysis (ICA).

**Methods:**

ICA-based analysis was performed on brain resting-state functional images of 19 volunteers.

**Results:**

We confirmed that DAN includes bilaterally: lobules VI-VII (crus I) and VIIB-VIIIA, as previously reported by Region-Of-Interest (ROI)-based functional connectivity studies. We also found that lobule IX (tonsillae), and as well as the superior and, likely, inferior colliculi. Also belong to DAN. The part of lobule IX in relation to DAN is located more caudally and laterally, and less extensive than the more rostral part of this lobule belonging to the default-mode network (DMN).

**Conclusion:**

Rostral and caudal tonsillae partake in the DMN and DAN, respectively. The latter could subserve either eye movement control in relation to the oculomotor parieto-frontal network, partially congruent with the DAN, or more cognitive functions due to functional reallocation within the DAN.

## Background

The cerebellum belongs to several major resting-state, intrinsically connected networks (default-mode, executive, salience, dorsal attentional and sensorimotor networks) (Fox et al. [1]; Habas et al. [2]; Buckner et al. [3]; Seng et al. [4]). Recently, using ROI-based functional connectivity at rest, and an attentional task-based fMRI paradigm, Brissenden et al. [5] demonstrated that cerebellar lobules VIIb and VIIIa also take part in the DAN, in line with Buckner et al. [3]. DAN is involved in environmental salient signal filtering, cue detection in relation to performance feed-back, subsequent (top-down) central attentional reallocation to salient or task-relevant stimuli, and working memory. This network largely overlaps with the circuit controlling (saccadic) eye movements (Corbetta et al. [6]) at the cortical level. However, at the cerebellar level, shifting attention to a peripheral target recruited posterior hemispheres of the lobule VI, whereas the saccades engaged the medial lobule VI. Shifting attention and orienting eyes to a salient peripheral stimulus are tightly and functionally linked. It could be assumed that the DAN phylogenetically derived from the circuit in charge of ocular saccades/smooth pursuit of a moving visual target, and became preferentially involved in higher cognition-monitored attentional processes without overt eye movement. Therefore, during the brain resting state, DAN could reflect both intermingled attentional and oculomotor circuits. Unexpectedly, Independent Component Analysis (ICA)-based functional connectivity did not report cerebellar contribution to DAN, to our knowledge.

The goal of the present study was first to identify cerebellar nodes of DAN, using ICA at rest, and consequently to try to replicate previous data obtained with ROI-based functional connectivity studies. Second, we wondered whether such cerebellar components would belong to the oculomotor or to the cognitive cerebellum, or both.

## Methods

### Subjects

Nineteen right-handed subjects (10 females; 9 males; mean age: 26.6; SE: 4.3) were scanned, while they remained still, motionless and kept their eyes closed. All of them provided their written informed consent, and had no history of cardiovascular nor neurologic disease.

### Acquisition sequences

Resting-state fMRI was performed on a whole-body 3 T scanner (Siemens, Skyra, Erlangen, Germany) with a sixty-four -channel head coil. Sixty contiguous axial multi-band SMS) T2*-weighted gradient echo-planar images (echo time 30 ms, repetition time 1000 ms, flip angle 90°, spatial resolution 2.5 × 2.5 × 2.5 mm, acceleration factor 2), were acquired to encompass the whole brain. Three hundred volumes were acquired with four “dummy” volumes recorded at the start of the one-run session to allow for steady-state magnetization.

### Post-processing

Group-level analysis (*N* = 19) was performed, using probabilistic, temporal concatenation-based Independent Component Analysis (ICA) implemented in the MELODIC software version 5.0.10, part of FSL (http://www.fmrib.ox.ac.uk/fsl).

Spatial ICA is a model-free, data-driven method which decomposes fMRI data into a set of component representing specific neural networks and noise-related patterns. During the brain resting-state, each component, or spatial map, includes brain areas whose spontaneous low-frequency BOLD fluctuations (0.01–0.1 Hz) are temporally correlated, and all the components are statistically independent (Beckmann et al. [7]).

Images were first preprocessed (head motion corrected applying rigid-body transformation (MCFLIRT), spatially smoothed with a 5-mm Full Width at Half Maximun (FWHM) kernel, default high pass temporal filtered, coregistered using a template brain of Montreal Neurological institute (MNI)). Head motion did not exceed 0.5 mm in x, y and z directions for all subjects. Second, these preprocessed data were « withened and projected into a 30-dimensional subspace using Principal Component Analysis (PCA)». Third, ICA analysis with a usual posterior probability threshold equal to 0.05, was carried out. Cluster localization was determined using probabilistic, cerebellum atlas for affine alignment after skull stripping (FLIRT) (http://www.diedrichsenlab.org/imaging/propatlas.html) and brain atlas provided by FSLview interface. We double-checked cluster location within the cerebellum resorting to Schmahmann et al.’ atlas [8]. Components were visually inspected and DAN was identified based on its well-known fronto-parieto-temporal anatomy clearly distinct from the other intrinsically connected networks.

## Results

### Functional data

**Fig. 1:**
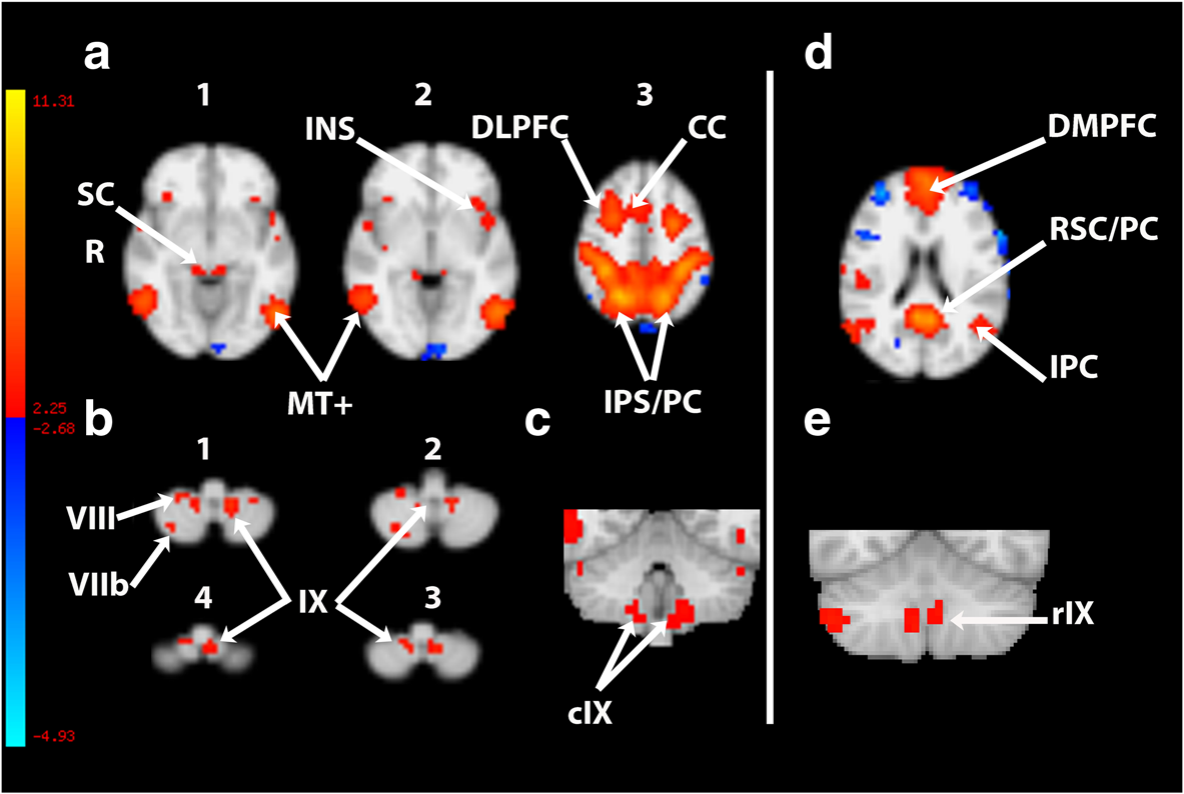
Multisubject spatial map (*N* = 19) of two components computed by ICA analysis and showing the dorsal attentional (**a1-a3, b1-b4, c**) and the default-mode networks (**d-e**). **a** axial slices passing through the encephalon. **b** and **e**. Axial and contiguous slices passing through the cerebellum. **c** and **d**. coronal slice passing through the cerebellum. CC, (para-) cingulate cortex; DLPFC, dorsolateral prefrontal cortex including caudally the frontal eye field; DMPFC, dorsomedian prefrontal cortex; INS, insula; IPS/PC, intraparietal sulcus/parietal cortex; MT+, medial temporo-occipital cortex; RSC/PC, retrosplenial cortex /precuneus; SC, superior colliculi. R, right. c, caudal; r, rostral. Latine numbers designate the cerebellar lobule. The bar from blue (negative) to yellow (positive) represents the z-value (percentage change of BOLD signal)

**Fig. 2:**
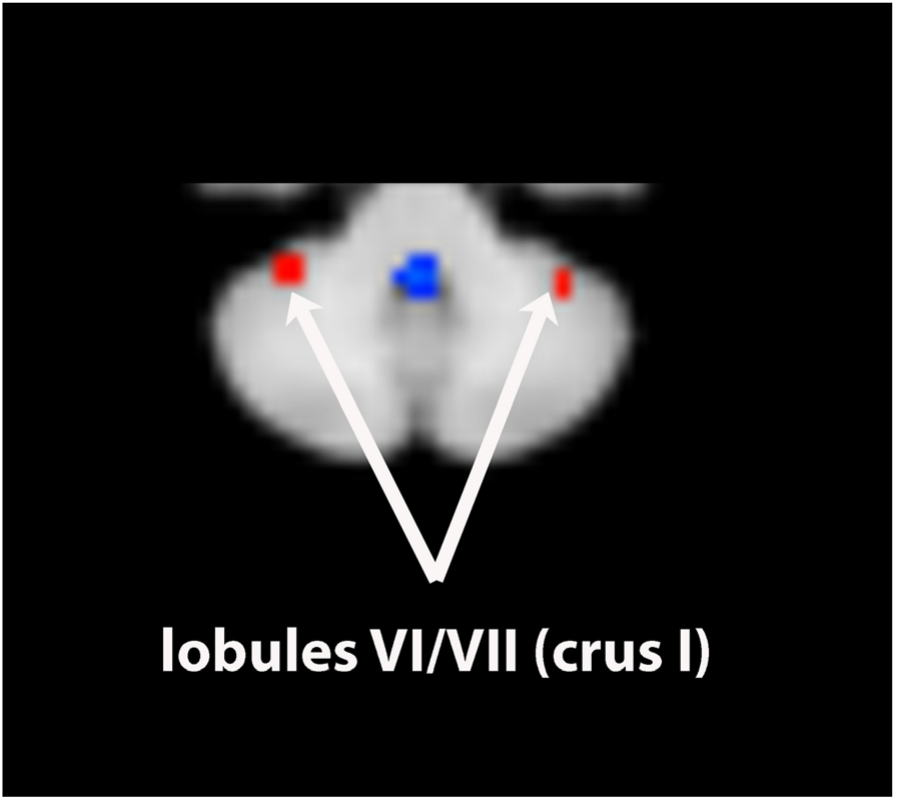
Axial slice passing through the cerebellum showing bilateral clusters located in lobules VI/VII (crus I)

The seventh ICA component corresponds to the dorsal attentional network including (Figs. 1a-d and 2): cerebellum (lobules VI/crus I with a right predominance, lobules VIIb with a right predominance, VIIIA/b, extending to the adjoining lateral and caudal IX, the so-called tonsillae, with a left predominance) (Table 1), superior colliculi, anterior insula with an extension to the frontal opercule, temporo-occipital cortex (likely MT+), the intraparietal sulcus with extensions to the adjoining superior and inferior parietal lobules, mid-cingulate cortex, and frontal eye field with a more rostral dorsolateral prefrontal cortex. We also considered the DMN (Component 1; Fig. 1e) which encompassses the rostral lobule IX bilaterally, without overlapping with the tonsillar part of the DAN.

**Table 1:** Cluster localization

		right side	left side
		(x, y, z)^a^	(x, y , z)^a^
Cerebellum	Lobules VI/VII (crus I)	37.81;-40.48;-30.03	− 35.79;-45.59;-30.03
	Lobule VIIb	32.70;-43.54;-49.53	− 30.68;-52.74;-49.53
	Lobule VIIIa/b	17.36; − 46.61;-57.64	− 18.41;-50.70;-57 .64
	Lobule IX	10.20;-52.74;-52.53	− 10.23;-51.72;-52.53

^a^MNI coordinates

The other well-delineated, intrinsically connected circuits comprised: visual (component 2), auditory (component 4), right executive (component 5), prefrontal (component 8), left executive (component 9), cerebellar (component 16), and sensorimotor (component 18) networks.

## Discussion

We first confirmed, using ICA, that lobules VIIb-VIIIA and ventral VI-crus I are parts of the DAN, in agreement with Buckner et al. [3] and Brissenden et al. [5]. We found additionnally that the lateral and caudal lobule IX, adjacent to lobule VIII, (also called tonsillae or ventral paraflocculus), and the superior colliculi, also belong to DAN, and that, consequently, caudal part of tonsilla must be differentiated from the rostral and medial lobule IX specifically in relation with the DMN.

First, lobule IX receives few afferents from the medial extrastriate occipital cortex via lateral pontine and reticular nuclei, in monkey (Glickstein et al. [9]) and gives off efferents to interposed, dentate and vestibular nuclei (Nagao et al. [10]). Moreover, this lobule is interconnected with the superior colliculi in the rat (Gayer and Faull [11]). Ablation of the ventral paraflocculus, in monkey, is accompanied by alteration of the vestibuloocular reflex and, mainly, of smooth pursuit eye movements (Rambold et al. [12]). Second, fMRI has detected, in human, tonsillar activation during horizontal optokinetic reflexive movement (Dietesrich et al. [13]), visually guided horizontal saccades (Stephan et al. [14]), error processing needed to maintain saccadic accuracy (Ettinger et al. [15]; Schraa-Tam et al. [16]) and spatial navigation task ascribed to the optic flow processing (Igloi K et al. [17]). This last hypothesis is in line with previous studies showing that the ventral paraflocculus received informations about displacement of the visual scene via pontine and pretectal nuclei (Kawano et al. [18]). Third, from a human clinical standpoint, lesions of tonsillae yield to head-shaking nystagmus (Huh and Kim [19]), nystagmus, and smooth pursuit and gaze-holding impairments (Lee et al. [20]). Hypometabolism was observed in tonsillae (and in flocculus) during downbeat nystagmus (Schlindwein et al. [21]) and likely in relation to desinhibition of the vestibular system. Tonsillae are thus implicated in monitoring and in feed-back-based adaptation of several types of eye movements. Fourth, Seng et al. [4] found out functional coherence between the vermal lobule IX (uvula) and the vision network. Consequently, the human lobule IX, or, at least, one subregion of it, conserves an important role in eye movements, so that it would be amazing that no functional coherence between tonsillae and oculomotor brain areas may exist. In this vein, the caudal dentate nucleus projects to the frontal eye field and to the posterior parietal cortex involved, for instance, in visual recalibration (area 7b in monkeys; Clower et al. [22]). Fifth, in our study, tonsillar clusters were located more caudally and laterally than the more rostral and medial tonsillar clusters associated with the DMN. Consequently, lobule IX must be specifically in functional coherence with the precuneus, part of the DAN and the network controlling saccade and smooth pursuit and likely with superior colliculi. In rat, interconnections between superior colliculi and ventral paraflocculus, through reticular/pontine nuclei have been traced (Gayer and Fauli [23]).

Lobule IX was shown to be a node of the DMN, as well as of the right executive network (Habas et al. [2]), and to be in functional coherence with caudate nuclei, hippocampus, amygdala (Seng et al. 2012 [4]), anterior cingulate cortex and the precuneus (Buckner et al. [3]), as well as with medial prefrontal cortex (Krienen and Buckner [24]; Bernard et al. [25]). It is noteworthy that lobule IX is predominantly functionally interconnected with the main brain areas belonging to the DMN. However, electrostimulation of the caudal precuneus can induce saccades in monkey (Thier and Andersen [26]). In cat, lobule IX was shown to receive afferents from mamillary nuclei and cingulate cortex and to give off Purkinje axons to the parvicellular dentate nucleus (Aas and Brodal [27]). This mamillary inputs could modulate tonsillar computation with motivational or spatial memory-based informations. In monkey, the same lobule is connected with middle prefrontal cortex (BA 46) (Kelly and Strick [28]) subserving, for instance, working memory. In our article [2], we also reviewed several sudies pointed out tonsillar activation during non ocular sensory, motor, emotional and higher cognitive tasks (thirst satiation, time perception, working memory, past and future event elaboration). Lobule IX is activated when participants observed negative emotion such as anger or disgust (Baumann and Mattingley [29]; Schraa-Tam et al. [30]) Importantly, Imamizu et al. [31] observed persistent tonsillar recruitment after tool manipulation learning, likely reflecting acquired internal model although the sensorimotor cerebellum corresponds to lobules V-VI and VIII. Therefore, lobule IX could participate in processing internal models for vision requiring compensations of eye dynamics during smooth pursuit, for example (Krauzlis, [32]), for non-visual predictive control (Kettner et al. [33]), and for more general (cognitive) purposes. Zwicker et al. [34] found an underactivation of the left lobule IX while children suffering from developmental coordination disorder, performed a trail-tracing task. In this case, lobule IX could be implicated in attention and/or in on-line eye-hand coordination. Furthermore, cortical atrophy of this lobule has been noted in autism spectrum disorder and in attention deficit hyperactivity disorder (Stoodley [35]). More recently, Guell et al. [36] found also tonsillar implication in langage and social processing. In summary, lobule IX appears to be; 1. preferentially interconnected with associative cognitive and limbic areas, 2. activated during executive and emotional processing, and 3. to be partly a site of internal model retention. Therefore, lobule IX could be regarded as a multimodal hub which interconnects three, sometimes anticorrelated (Fox et al. [1]), networks: DAN, executive network and DMN.

However, we emphasize that if we agree that tonsillae should « fall within tertiary nonmotor representation map» as suggested by Buckner et al. [3] and Guell et al. [36], it must also implement an oculomotor representation according to clinical data and to some aforementioned fMRI studies (Voogd et al. [37]). We postulated that clusters within lobule IX, we detected, could either reflect an oculomotor network embedded in DAN, or functional reallocation of tonsillae to attentional processing, or also tonsillar contribution to both circuits. Putatively and from a phylogenetical standpoint, the lobule IX may have been involved first in the control and the adaptation of eye movement such as saccades and smooth pursuit. In a second time with the telencephalization process, the DAN might have partially differenciated from the cerebello-fronto-(temporo)-parietal monitoring eye movements, so that lobule IX, in conjunction with the adjoining lobules VII-VIII, might have also been implicated in top-down attentional processes. This anatomical and functional process should have also concerned the superior colliculi and the precuneus connected with tonsillae. Left lobule IX might have partly been ascribed to visuo-spatial processing in the right executive control network. Lastly, the major part of the lobule IX has been incorporated into the DMN.

Further studies are thus required to test these hypotheses, especially to test anatamo-functional parcellation of lobule IX, using ROIs-based functional connectivity and tractography seeding different sub-regions of the lobule IX. Moreover, we used FLIRT (FMRIB’s Linear Image Registration Tool) option to coregister the functional mean image with the brain MNI template. However, non-linear methods of coregistration, such as FSL’s FNIRT and especially SUIT (Spatially Unbiased Infratentorial Template), for the cerebellum, give better and more accurate alignement, and should be preferred to linear methods. The main problem is the spatial spead of cerebellar fissures exposing to an important risk of lobular mislocation. However, the large cerebellar amygdalae (lobule IX), and especially their medial part, can be well-delineated, and the risk of mislocation may predominantly concern their lateral part close to the secundary fissure, i.e. the junction between lobules VIIIB and IX. In the current study, most of the voxels within caudal tonsillae were situated far from the secundary fissures, and might not have mislocated.

Finally, if the lobules VII, VIII and IX are the main cerebellar zones included in DAN, as noted earlier, lobules VI/crus I also but to a lesser extent contribute to DAN. These latter lobules are in functional coherence with the prefrontal cortex, and partake in executive networks. However, if these lobules can reflect functional association between DAN and prefronto-cortical nodes of executive control network, they can also belong to oculomotor control networks (Voogd et al. [37]).

## Conclusion

Caudal and rostral regions of the lobule IX (tonsillae) contribute to the anticorrelated DAN and DMN, respectively, whereas the left tonsilla takes part in the right executive control network. However, caudal lobule IX could also reflect an oculomotor circuit embedded in the DAN and specifically devoted to oculomotor error tracking and correction.

## Abbreviations

DAN: Dorsal attentional network
DMN: Default-mode network
