# Correction to: Participation of the caudal cerebellar lobule IX to the dorsal attentional network

**DOI:** 10.1186/s40673-018-0089-7

**Published:** 2018-08-02

**Authors:** Stephen Ramanoel, Elizabeth York, Christophe Habas

**Affiliations:** 1 Institut de la Vision (CHNO des 15-20), CNRS, INSERM, Université Pierre et Marie Curie, 28, rue de Charenton, 75012 Paris, France; 2 Service de NeuroImagerie, CHNO des 15-20, Paris, France

## Correction

After publication of the original article [1], the authors reported their Given Names and Family names had been incorrectly reverted so they were presented as: Ramanoel Stephen, York Elizabeth and Habas Christophe. The correct presentations of the authors’ names are included in this correction. The original article has been corrected.
